# The long-term efficacy of one-shot neoadjuvant intra-arterial chemotherapy combined with radical cystectomy versus radical cystectomy alone for bladder cancer: a propensity-score matching study

**DOI:** 10.1186/s12894-019-0552-7

**Published:** 2019-11-16

**Authors:** Wasilijiang Wahafu, Sai Liu, Wenbin Xu, Mengtong Wang, Qingbao He, Liming Song, Mingshuai Wang, Feiya Yang, Lin Hua, Yinong Niu, Nianzeng Xing

**Affiliations:** 1 Institute of Urology, Capital Medical University, Department of Urology, Capital Medical University Beijing Chao-Yang Hospital, Beijing, 100020 China; 2 Department of Urology, National Cancer Center/National Clinical Research Center for Cancer/Cancer Hospital, Chinese Academy of Medical Sciences and Peking Union Medical College, Beijing, 100021 China; 3 School of Biomedical Engineering, Capital Medical University, Beijing, 100069 China

**Keywords:** Bladder cancer, Neoadjuvant chemotherapy, Intra-arterial infusion, Cystectomy, Treatment outcome

## Abstract

**Background:**

Bladder cancer is a complex disease associated with high morbidity and mortality. Management of bladder cancer before radical cystectomy continues to be controversial. We compared the long-term efficacy of one-shot neoadjuvant intra-arterial chemotherapy (IAC) versus no IAC (NIAC) before radical cystectomy (RC) for bladder cancer.

**Methods:**

We performed a retrospective review of patients who underwent either one-shot IAC or NIAC before RC between October 2006 and November 2015. A propensity-score matching (1:3) was performed based on key characters. The Kaplan-Meier method was utilized to estimate survival probabilities, and the log-rank test was used to compare survival outcomes between different groups. A multivariable Cox proportional hazard model was used to estimate survival outcomes.

**Results:**

Twenty-six patients were treated using IAC before RC, and 123 NIAC patients also underwent RC. After matching, there was no significant difference between groups in baseline characteristics, perioperative variables, complication outcomes or tumor characteristics. Compared with clinical tumor stages, pathological tumor stages demonstrated a significant decrease (*P* = 0.002) in the IAC group. There was no significant difference in overall survival (OS, *p* = 0.354) or cancer-specific survival (CSS, *p* = 0.439) between the groups. Among all patients, BMI significantly affected OS (*p* = 0.004), and positive lymph nodes (PLN) significantly affected both OS (p<0.001) and CSS (*p* = 0.010).

**Conclusions:**

One-shot neoadjuvant IAC before RC shows safety and tolerability and provides a significant advantage in pathological downstaging but not in OS or CSS. Further study of neoadjuvant combination therapeutic strategies with RC is needed.

## Background

Bladder cancer is a complex disease associated with high morbidity and mortality rates. Approximately 75% of newly diagnosed patients present with non-muscle-invasive bladder cancer (MIBC), which is characterized by a high recurrence rate and 5-yr survival of ~ 90% [1]. Once the disease becomes MIBC, the 5-year overall survival is a dismal outcome at 47% compared with the 81% survival rate of patients with non-muscle-invasive disease [2]. Approximately 50% of MIBC patients will develop metastasis and have a 5-yr survival of only ~ 5% [3, 4]. Despite radical cystectomy (RC) with bilateral pelvic lymph node dissection (PLND) as the gold standard treatment, RC only permits a 5-yr survival in approximately 50% of patients [3, 5–8]. In fact, there was no significant improvement in bladder cancer outcomes over the last three decades.

Although several high-quality clinical trials have demonstrated improved survival and pathologic downstaging with the use of chemotherapy prior to RC, adoption of neoadjuvant chemotherapy for MIBC has been slow. Several hypotheses, such as the significant toxicities and delayed surgery, especially the inability to identify which patients could derive the most benefit from neoadjuvant chemotherapy, were slow during the adoption of neoadjuvant treatment. Additionally, 25 to 33% of patients are unable to receive adjuvant chemotherapy after RC due to postoperative problems, such as perioperation complications or deterioration of renal function [9, 10]. Therefore, we hypothesized that one-shot neoadjuvant intra-arterial chemotherapy (IAC) would have less toxicity and better disease control than RC alone. Moreover, this strategy would allow patients to complete therapy quickly and move on to the next form of therapy.

Therefore, we compared the long-term efficacy of one-shot neoadjuvant IAC versus no IAC (NIAC) before RC for bladder cancer in this study.

## Methods

To evaluate the long efficacy of one-shot neoadjuvant IAC versus NIAC before RC for bladder cancer, we retrospectively reviewed all patients treated with RC/PLND between October 2006 and November 2015 for urothelial carcinoma of the bladder without distant metastasis in the Department of Urology, Beijing Chao-Yang Hospital. This study was approved by the Institutional Review Board of Beijing Chao-Yang Hospital. To prevent selection bias of the learning curve, we chosen patients who operations were performed by the same laparoscopic surgeon (Xing).

### Patient eligibility and selection

The diagnosis of bladder cancer was made using imaging findings (ultrasonography, computed tomography, magnetic resonance imaging), chest radiography with or without cystoscopic biopsy, and routine laboratory analysis. The TNM classification was staged according to the American Joint Committee on Cancer staging system (7th 2010). Clinical staging was based on the physical examination, imaging findings, and biopsies of bladder tumors before the start of therapy. All patients had pathologic documentation of urothelial carcinoma, which was defined as local disease (pT2-4 N0/+M0) or non-muscle-invasive bladder cancer (NMIBC), but the patients were at high risk for tumors [T1G3 with concurrent carcinoma in situ (CIS) at diagnosis, multiple and/or large T1G3, recurrent T1G3]. The pathological results were reviewed by the two genitourinary pathologists after matching the two groups. Patient with pelvic lymph node metastasis diagnosed by imaging studies were eligible. Patients who underwent neoadjuvant intravesicle chemotherapy but not adjuvant chemotherapy were ineligible. Patients who had nonurothelial carcinoma (*n* = 11), preoperative pelvic irradiation (*n* = 5), missing clinical information (n = 11) or who were lost during follow-up (*n* = 17) were excluded, leaving 149 patients available for analysis.

### IAC treatment protocol

Gemcitabine (700–1000 mg/m^2^) and cisplatin (35–70 mg/m^2^) were infused into the femoral artery to the internal iliac artery using the Seldinger technique. The approach of 15 patients was from the bilateral internal iliac artery, while the unilateral internal iliac artery was used in 11 patients, and the approach was based on tumor location as determined by imaging tests, cystoscopy and digital subtraction angiography. Complete blood counts and biochemical studies were performed every 2 weeks. Patients were evaluated for treatment responses using imaging tests and were assigned to receive RC/PLND 4 weeks after IAC to allow adequate recovery.

### Statistical analysis

#### Baseline comparison between the intra-arterial and no intra-arterial groups

Key baseline characteristics [gender, age, Body mass index (BMI), hypertension, diabetes, age-adjusted Charlson comorbidity index (CCI), American Society of Anesthesiologists (ASA) score, Eastern Cooperative Oncology Group performance status (ECOG PS), smoking history, time between tumor confirmation and RC, preoperative irradiations, and follow-up duration)]were compared between the IAC and NIAC groups.

Continuous characters were compared by independent sample t-tests when the data were normally distributed and by Wilcoxon rank sum test when the data were nonnormally distributed. The Pearson chi-square test or Fisher’s exact test was performed to calculate *p* values for categorical factors. The Wilcoxon rank sum test was performed to compare ordinal values.

#### Propensity-score matching

We performed matched group analysis to control for differences between groups due to selection bias and confounding factors. Propensity-score matching was performed based on key characters, including gender, age, BMI, hypertension, diabetes, age-adjusted CCI, ASA score, ECOG PS, smoking history, time between tumor confirmation and RC, preoperative irradiations and follow-up duration. Propensity scores were estimated using a logistic regression model. A 1:3 matching with no replacement was applied using the nonrandom package in R (http://www.r-project.org). A t-test or Wilcoxon rank sum test, or Pearson’s chi-square test or Fisher’s exact test, was applied to compare differences in covariates after matching to demonstrate that matching enhanced the balance between groups.

#### Oncological outcomes in the matched group

We compared oncological outcomes in a matched cohort using a t-test, a Wilcoxon rank sum test, Pearson’s chi-square tests and Fisher’s exact test. The Kaplan-Meier method was utilized to estimate survival probabilities, and the log-rank test was used to compare survival outcomes between different groups. A multivariable Cox proportional hazard model was used to estimate survival outcomes.

All statistical analyses, except for propensity-score matching, were performed with IBM SPSS version 19.0 (IBM corp., Armonk, NY). Statistical significance was considered at two-sided *p* < 0.05. All statistical plots were drawn in GraphPad prism version 6.0 (GraphPad Software Inc., La Jolla, CA 92037 USA).

## Results

A total of 26 patients underwent one-shot neoadjuvant IAC, and 123 patients were treated using RC/PLND alone. The baseline characteristics of patients enrolled are listed in Table 1. All key variables except follow-up duration (88 mo vs 26 mo, *p* = 0.002) were not different at baseline between the two groups. To reduce the differences between groups due to selection bias, we performed a matched analysis based on follow-up duration Additional file 2 Figure S1.

**Table 1:** Baseline characteristics of the patients in the IAC and NIAC before and after matched groups (1:3)

	Intra-arterial	Before matched groups	After matched groups (1:3)		
		No intra-arterial	p value	No intra-arterial	p value
Patients (n)	26	123		78	
Gender			1.000		1.000
Female, n (%)	4(15.4%)	19(15.4%)		10(12.8%)	
Male, n (%)	22(84.6%)	104(84.6%)		68(87.2%)	
Age, yr, median (IQR)	60.0(55.0–71.0)	63.0(56.0–72.0)	0.328	62.5(56.0–69.3)	0.799
Body mass index (kg/m2)	25.2 ± 3.12	24.1 ± 3.8	0.184	24.3 ± 3.1	0.202
Hypertension, n (%)	12(46.2%)	38(30.9%)	0.134	27(34.6%)	0.293
Diabetes, n (%)	4(15.4%)	16(13.0%)	0.995	8(10.3%)	0.723
Age-adjusted CCI	4.0(3.0–7.0)	4.0(3.0–5.0)	0.625	4.0(3.0–6.0)	0.909
ASA score	2.0(1.8–2.0)	2.0(2.0–2.0)	0.221	2.0(2.0–2.0)	0.188
ECOG PS	1.0(0.0–1.0)	1.0(0.0–1.0)	0.490	1.0(1.0–1.0)	0.394
Smoking history, n (%)	15(57.7%)	58(47.2%)	0.329	40(51.3%)	0.571
Time between confirmed tumor and RC, mo, median (IQR)	3.0(1.0–6.8)	5.0(1.0–18.0)	0.133	5.0(1.0–18.0)	0.173
TURBT before RC	7(25.9%)	57(46.3%)	0.048	32(41.0%)	0.100
Preoperative irradiation, n (%)	0(0.0%)	5(4.1%)	0.587	3(3.8%)	0.571
Follow-up length, mo, median (IQR)	88.0(37.0–109.0)	26.0(14.0–65.0)	0.002	56.0(30.8–91.3)	0.161

IAC, intra-arterial chemotherapy; NIAC, no-intra-arterial chemotherapy; IQR = interquartile range; RC = radical cystectomy; ASA = American Society of Anesthesiologists; CCI = Charlson comorbidity index; ECOG PS = Eastern Cooperative Oncology Group performance status

The matching algorithm was 1:3, which was the optimal weight for each key variable. The patients were followed up for a median period of 88 months in the IAC group and for 56 months in the NIAC group (*p* = 0.161). There were no significant differences between the groups in patient demographics and clinical characteristics. Table 1 lists the baseline characteristics for the matched cohorts.

There was no significant difference in perioperative variables between the IAC and NIAC groups (Table 2). In the type of urinary diversion, more than 50% of patients received orthotopic neobaldders in both groups. IAC treatment did not affect renal function in terms of serum creatinine (*P* = 0.702) or blood urea nitrogen (*P* = 0.119) levels. The proportion of those who remained in the intensive care unit after surgery was lower in the IA group than in the NIAC group (0% vs 10.3%; *p* = 0.196). The total complication rate was not significantly different between the two groups (92.3% vs 96.2%; *p* = 0.791). However, Clavien grade 2 complications (>80%) were more common in the perioperative period (< 30 d).

**Table 2:** Perioperative variables of the matched groups

	Intra-arterial	No Intra-arterial	p value
Patients (n)	26	78	
Type of urinary diversion, n (%)			0.840
Cutaneous ureterostomy	2(7.7%)	5(6.4%)	
Ileal conduit	9(34.6%)	32(41.0%)	
Orthotopic neobladder	15(57.7%)	41(52.6%)	
Operating time, min, mean (IQR)	369.0(300.0–420.0)	382.9(306.0–420.0)	0.574
Estimated blood loss, ml, mean (IQR)	411.5(187.5–525.0)	348.1(200.0–400.0)	0.456
Removed Jackson-Pratt drain, day, mean (IQR)	12.6(9.0–14.3)	14.7(8.0–19.0)	0.591
Passing flatus, day, mean (IQR)	4.9(3.0–6.0)	4.0(3.0–5.0)	0.189
Adjuvant chemotherapy, n (%)	4(15.4%)	12(15.4%)	1.000
Pre-op laboratory studies			
HGB (g/L), median (IQR)	134.0(122.3–142.3)	132.5(119.8–146.3)	0.943
HCT (%), median (IQR)	38.6(36.9–41.5)	39.8(36.1–42.4)	0.615
WBC, median (IQR)	6.4(5.0–7.6)	6.5(5.3–7.8)	0.286
Platelets, median (IQR)	218.5(193.0–262.5)	216.5(187.3–258.5)	0.768
BUN(mmol/L), median (IQR)	5.7(4.7–7.0)	6.1(4.6–8.0)	0.119
Creatinine(μmol/L), median (IQR)	84.2(70.3–113.5)	82.2(70.1–99.9)	0.702
Albumin (g/L), median (IQR)	35.1(32.8–39.0)	36.0(33.0–39.9)	0.931
Overall complications, n (%), Clavien grade	24(92.3%)	75(96.2%)	0.791
Perioperative complications (< 30 d), n (%),			0.930
0	2(7.7%)	3(3.8%)	
1	0(0.0%)	2(2.6%)	
2	21(80.8%)	66(84.6%)	
3	3(11.5%)	5(6.4%)	
4	0(0.0%)	0(0.0%)	
Short-term complications (< 90 d), n (%)			0.516
0	24(92.3%)	68(87.2%)	
1	0(0.0%)	4(5.1%)	
2	1(3.8%)	3(3.8%)	
3	1(3.8%)	3(3.8%)	
4	0(0.0%)	0(0.0%)	
Long-term complications (>90 d), n (%)			0.616
0	24(92.3%)	74(94.9%)	
1	0(0.0%)	0(0.0%)	
2	0(0.0%)	0(0.0%)	
3	1(3.8%)	3(3.8%)	
4	1(3.8%)	1(1.3%)	
Surgery intensive care unit stay, n (%)	0(0.0%)	8(10.3%)	0.196

IQR = interquartile range; HGB = hemoglobin; HCT = hematocrit; WBC = white blood cell; BUN = blood urea nitrogen

Tumor characteristics are listed in Table 3. The pathology results of all patients showed urothelial cell carcinoma of the urinary bladder. Positive surgical margins were reported in the NIAC group (3.8%). Compared with clinical TNM stages, pathological TNM staging demonstrated similar in the NIAC group after matching (*P* = 0.519, Additional file 1 Table S1 and Additional file 2 Figure S2); however, a significant decrease showed in the IAC group (*P* = 0.002): 7 (26.9%) patients had no stage change, 17 (65.4%) patients exhibited a stage decrease, and 2 (7.7%) patients exhibited a stage increase (Additional file 1 Table S2 and Additional file 2 Figure S3). There was one patient with severe gross hematuria that was diagnosed as NMIBC by CT. Conservative measures and attempts to achieve hemostasis by cystoscopy were unsuccessful at controlling bleeding. The patient therefore underwent endovascular treatment with intra-arterial chemotherapy and superselective embolization of the vesical arteries 2 weeks before RC/PLND.

**Table 3:** Tumor characteristics of the matched groups

	Intra-arterial	No intra-arterial	p value
Patients (n)	26	78	
Pathologic stage outcome, n (%)			0.414
pT1	9(34.6%)	26(33.3%)	
pT2a	6(23.1%)	11(14.1%)	
pT2b	1(3.8%)	14(17.9%)	
pT3a	6(23.1%)	11(14.1%)	
pT3b	3(11.5%)	3(3.8%)	
pT4a	1(3.8%)	13(16.7%)	
Histology grade, n (%)			0.566
Low grade	6(23.1%)	14(17.9%)	
High grade	20(76.9%)	64(82.1%)	
Pathology, n (%)			0.399
Urothelial cancer	21(80.8%)	71(91.0%)	
Urothelial cancer with squamous differentiation	3(11.5%)	4(5.1%)	
Urothelial cancer with glandular differentiation	2(7.7%)	3(3.8%)	
Nodes removed, median (IQR)	17.0(11.8–21.3)	14.0(8.0–19.0)	0.304
PLN, median (range)	0.0(6.0)	0.0(27.0)	0.904
Lymph-node-positive patients, n (%)	7(26.9%)	18(23.1%)	0.691
Positive surgical margins, n (%)	0(0.0%)	3(3.8%)	0.571
Associated CIS, no. (%)	4(15.4%)	12(15.4%)	1.000

IQR = interquartile range; CIS = carcinoma in situ; PLN = positive lymph nodes

**Fig. 1:**
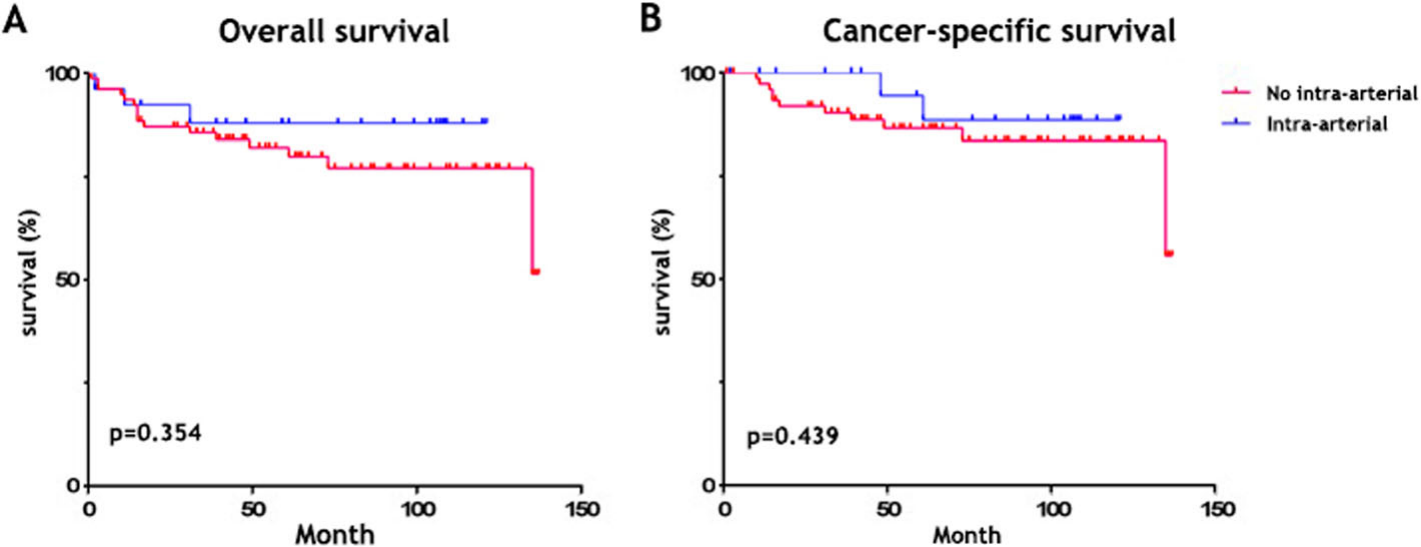
Overall survival and cancer-specific survival. **a** Three (11.5%) and sixteen (20.4%) patients died in the IAC and NIAC groups, respectively (*p* = 0.354). **b** Two (7.7%) and eleven (14.1%) patients suffered cancer-specific mortality in the IAC and NIAC groups, respectively (*p* = 0.439)

Of the 26 patients in the IAC group, two (7.7%) died because of cancer, and one (3.8%) died due to another reason. Among the 78 patients in the NIAC group, eleven (14.1%) suffered cancer-specific mortality, and five (6.4%) died due to another reason. There was no significant difference in the rates along the curve for overall mortality (*p* = 0.354) or cancer-specific mortality (*p* = 0.439) between the IAC and NIAC groups (Fig. 1).

Multivariable Cox proportional hazards regression analysis (Table 4) showed that several variables have an impact on overall survival. In all samples, BMI (*p* = 0.005), diabetes (*p* = 0.002), ASA score (p = 0.005), PLN (p<0.001) and perioperative complications (*p* = 0.020) were influencing factors.

**Table 4:** Multivariable Cox proportional hazards model to estimate survival outcomes

Variables	Total	
	p value	RR(95%CI)
BMI	0.005	0.767(0.638–0.922)
Diabetes	0.002	8.716(2.263–33.563)
ASA score	0.005	4.846(1.600–14.682)
Positive lymph nodes	<0.001	11.886(3.912–36.119)
Perioperative complication	0.020	4.416(1.259–15.488)

**Fig. 2:**
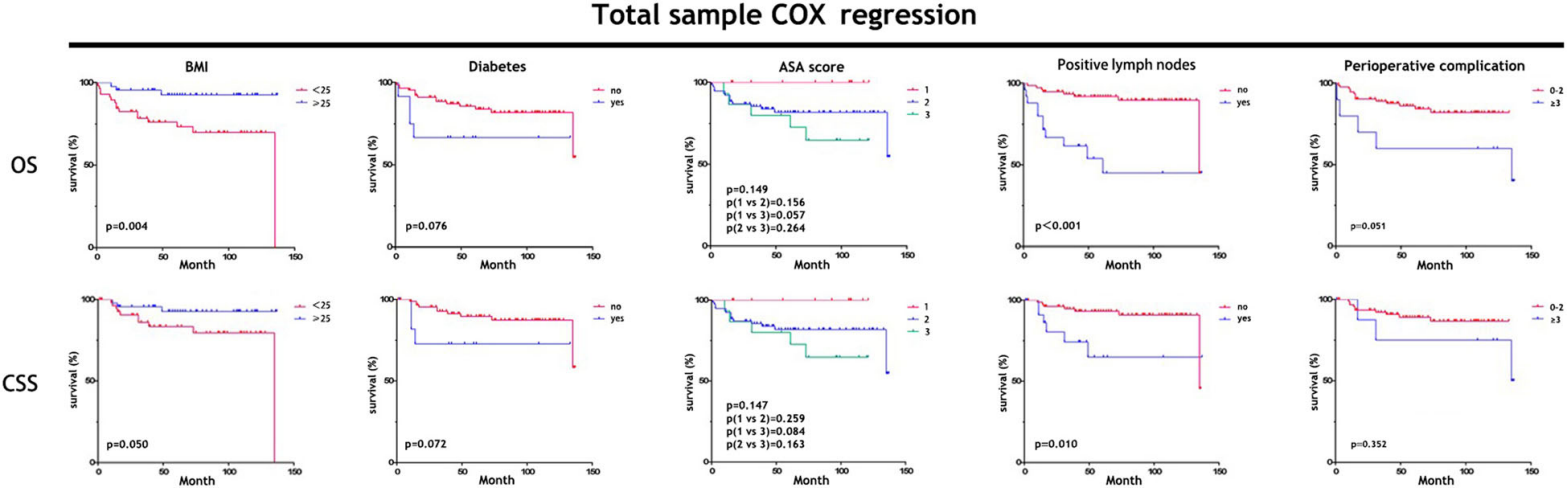
Overall survival and cancer-specific survival from Cox proportional hazards regression analysis (see Table 5). BMI less than 25 kg/m^2^ was associated with OS (*p* = 0.004) but not CSS (*p* = 0.050), and PLN was associated with OS (p<0.001) and CSS (*p* = 0.010) in all sample groups

When these potential factors were used to calculate the Kaplan-Meier survival curve, some were associated with OS and CSS (Fig. 2). BMI less than 25 kg/m^2^ was associated with OS (*p* = 0.004) but not CSS (*p* = 0.050), and PLN was associated with OS (p<0.001)and CSS (*p* = 0.010). The survival time and cumulative survival rate (1-, 5- and 10-year rates) are depicted in Table 5.

**Table 5:** Description of survival of groupings in the entire set of patients (see Fig. 2)

	Mean ST (mo)	Medium ST (mo)	1-year CSR (95%CI)	5-year CSR (95%CI)	0-year CSR (95%CI)
OS of BMI grouping					
<25	102.35	135.00	0.897(0.784–0.952)	0.733(0.587–0.835)	0.699(0.541–0.811)
≥25	129.00	–	0.956(0.0–1.0)	0.927(0.0–1.0)	0.927 (0.0–1.0)
OS of PLN grouping					
No	124.81	135.00	0.975(0.903–0.994)	0.920(0.830–0.963)	0.897(0.793–0.951)
Yes	75.65	61.00	0.800(0.000–1.000)	0.540(0.002–0.943)	0.450(0.006–0.884)
CSS of PLN grouping					
No	126.37	135.00	0.987(0.913–0.998)	0.932(0.843–0.971)	0.909(0.804–0.959)
Yes	98.10	–	0.861(0.000–1.000)	0.649(0.000–0.985)	0.649(0.000–0.985)

ST: survival time; CSR: cumulative survival rate; OS: overall survival; CSS: cancer-specific surival; BMI: body mass index; PLN: positive lymph nodes

## Discussion

Our present results show that there was a downstaging advantage with one-shot neoadjuvant IAC before RC for MIBC (p = 0.002), but it did not significantly improve OS (*p* = 0.354) or CSS (*p* = 0.439) compared to those treated without IAC. We performed Cox regression to assess risk factors association with survival in all samples and found that BMI (less than a 25 kg/m^2^) significantly affected OS (p = 0.004), and PLN significantly affected both OS (p<0.001 = and CSS (p = 0.010). Besides, we are curious about the potential risk factors affecting survival outcomes in the IAC and NIAC group and their difference in the two groups. So, despite the small sample size of IAC and NIAC group, we used Cox regression to explore the risks in the both groups exploratorily. The exploratory analysis found that diabetes (*P* = 0.029, RR = 14.649) was an influencing factor in IAC group, whereas BMI (*P* = 0.015, RR = 0.802), PLN (*P* < 0.001, RR = 7.474) and smoking history (*P* = 0.043, RR = 3.388) were influencing factors in NIAC group (Additional file 1 Table S3). Furthermore, when these potential factors were used to calculate the Kaplan-Meier survival curve, some were associated with OS and CSS in IAC groups and NIAC groups (Additional file 1 Table S4-S7 and Additional file 2 Figure S4). In brief, one-shot neoadjuvant resulted in significant downstaging; for RC, only BMI and PLN correlate with survival in our long-term data.

RC usually occurs 4 to 6 weeks after MIBC diagnosis in our center, and this time offers an opportunity to preoperatively perform neoadjuvant therapy. Although standard neoadjuvant cisplatin-based combination chemotherapy followed by RC is supported by level 1 evidence for resectable (cT2-T4aN0M0) MIBC, the inability to identify which patients may derive most benefit from neoadjuvant chemotherapy was slow during the adoption of neoadjuvant treatment. Nevertheless, approximately 50% of patients with urothelial carcinoma are considered ineligible to receive cisplatin based on renal dysfunction and impaired performance status, and a subset of patients also refuse to receive neoadjuvant chemotherapy [11]. Notably, adherence to adjuvant and neoadjuvant chemotherapy regimens was observed in a similarly low proportion of patients (approximately 21% each) in the USA, and the majority of patients with resectable bladder cancer received no chemotherapy at all [12]. Therefore, the treatment algorithm for MIBC tumors in a short window before RC is still evolving.

Neoadjuvant IAC is not a new concept. In the 1980s–1990s, multiple efforts were made to improve oncological outcomes by adding various IAC treatment modalities plus RC to treatment regimens for MIBC. A summary of the published neoadjuvant IAC papers, including key information on chemotherapy regimens, is provided in Table 6 (Additional file 2 Figure S5) [13–21]. Although most of the literature is early in its use, the drugs also have differences, but all show varying degrees of pathological downstaging or even complete response (CR; pT0). Pathological downstaging or pathological CR to neoadjuvant chemotherapy is a well-recognized biomarker of improved OS [22]. Because it was such a short period of therapy, we felt that achieving pathological CR would be quite challenging in our study. Although OS and CSS for the study cohort remained disappointing, one-shot neoadjuvant IAC showed an encouraging pathological downstaging rate of greater than 60% (*P* = 0.002). Meanwhile, the safety and tolerability profile for IAC was quite favorable. In particular, no chemotherapy-related adverse events have been reported in the IAC group, which did not delay planned surgery. Moreover, no differences in perioperative, short-term or long-term complications were recorded compared with patients undergoing RC only. Similarly, intraoperative performance (operating time, estimated blood loss, blood transfusion, number of nodes removed and surgical margins) was not compromised by neoadjuvant IAC. Therefore, our treatment produced major pathologic responses, indicating that the side effects of chemotherapy can be reliably avoided when using one-shot IAC.

**Table 6:** Summary of the published papers on neoadjuvant intra-arterial chemotherapy followed by radical cystectomy

Study	Year	Country	Type of study	Sample size (RC/total)	Chemotherapy regimen	No. of cycles	Interval to RC, (wks.)	Downstaging, (%), only RC	OS (only RC)
Kanoh et al. [13]	1983	Japan	Retrospective	7/13	ADM	2/wk. (≥3 wks)	6.7	5 (71.4)	2 died (14.6)
Kamidono et al. [14]	1984	Japan	Retrospective	11/11	ADM, MMC	1	4.2	7 (63.6)	3 died (17.5)
Maatman et al. [15]	1986	Italia	Prospective	16/25	CDDP, ADM	1–4	4	4 (25)	1 died (15.7)
Kanoh et al. [16]	1987	Japan	Retrospective	15/32	ADM ± CDDP	10–23 (17)	–	–	1 died, 5-year OS 87.5%
Kakizaki et al. [17]	1987	Japan	Retrospective	29/29	MMC, CPM, thio-TEPA, 5-FU, ADM, CDDP	1	2	–	–
Jacobs et al. [18]	1989	USA	Retrospective	16/30	CDDP	1	4	15 (93.8)	3 N+ average 13 mo 8 N0 average 28 ± 8 mo
Galetti et al. [19]	1989	USA	Phase II	4/8(only IA)	CDDP	1	–	3 (75)	37mo (6–56)
Arima et al. [20]	1997	Japan	Retrospective	80/120	ADM + CDDP	1–4	–	75 (62.5)	–
Miyata et al. [21]	2015	Japan	Retrospective	17/50	CDDP, ADM, EPI	2 ± 0.2	4–8	–	–
Recent study	2019	China	Retrospective	26/26	GC	1	4	17 (65.4)	3 died (2 from cancer: 11 and 31mo)

RC, radical cystectomy; OS, overall survival; ADM, adriamycin or doxorubicin; MMC, mitomycin C; CDDP, cisplatin; EPI, epirubicin; GC, gemcitabine + cisplatin; −, not available

Bladder cancer is a heterogeneous disease, which means that only single treatment is not enough. Current research is actively exploring novel combinations and ideal sequencing with various treatment modalities, especially immunotherapy combined with chemotherapy, radiotherapy or targeted therapies. Although bladder cancer carries the third highest mutation rate of all studied cancers, suggesting the possibility of increased immunogenicity via the development of neoantigens, it is clear from existing data that the majority of patients will not respond to monotherapy [23–25]. Interestingly, chemotherapeutic agents have direct cytotoxic effects on tumor cells that release tumor antigens but also have positive effects on immune effector T cells [26]. Therefore, in theory, one-shot IAC can play a synergistic role as a single immunotherapy. Moreover, chemotherapy may substantially prolong the total duration of neoadjuvant immunotherapy [27]. However, patient selection must be optimized. In addition to having good renal function, it is also necessary to pay attention to the patient’s nutritional status and immune system, which may be hampered by an aged-related reduction in functional decline. With an average age of 73 years at diagnosis, perioperative immunonutrition has a significant impact on surgery and the efficacy of immunotherapy [28]. According to our findings, BMI, diabetes and ASA score were associated with survival and may be the modifiable predictors in older and sicker patient populations. Additionally, the optimization of toxicity and tolerability of combination therapies through appropriate dosing and sequencing should be determined using well-designed clinical trials.

The strengths of our study are the selection of only one surgeon’s cases for minimizing the influence of different levels of maturity and the use of propensity-score matching to reduce the inherent biases. As a result, patients who were matched only on the basis of key variables were selected. However, an important limitation is our drawing conclusions from small sample and highly selected patients with retrospective, nonrandomized data, which might introduce possible selection biases that we did not control for. Another limitation of the present study was that there was no consistent record of recurrence-free survival (RFS) in the long-term follow-up period. Although the final pathology showed no difference between the cohorts, the proportion of positive surgical margins was higher in NIAC cohort. It is possible that NIAC cohort had lower stage disease to begin with which would affect the RFS of patients. However, it should be noted that OS is the gold standard and the most dependable end point in clinical cancer research to support treatment algorithms. Furthermore, CSS may be a surrogate endpoint for RFS. Nevertheless, we were not able to detect statistically significant differences between the groups in OS or CSS. At the same time, more than half of our patients were from all over the country, and some proportion of patients did not have clear data on disease recurrence. Therefore, RFS is not as important. Finally, we should know that there was not a specific marker to judge the safety, tolerability, or clinical benefit of the treatments in the subgroups of patients. Answers to some of these questions will become clearer as these studies begin to mature with clinical readouts.

## Conclusions

This long-term follow-up, retrospective study of one-shot neoadjuvant IAC in patients who underwent RC from 2006 to 2015 shows significant advantages in pathological downstaging but not in OS or CSS. Moreover, this study demonstrates the safety and tolerability of this treatment and provides a basis for combination therapy. Future efforts to improve survival in patients with bladder cancer is warranted and further study of the ideal neoadjuvant therapeutic strategies followed by RC is needed.

## Supplementary information

**Additional file 1: Table S1.** Pathological staging before and after surgery in the NIAC group after matching (see Fig. S2), **Table S2.** Pathological staging before and after surgery in the IAC group (see Fig. S3), **Table S3.** Multivariable Cox proportional hazard model to estimate survival outcomes in IAC and NIAC groups, **Table S4.** Description of OS of diabetes groupings in the IAC group (see Fig. S4A), **Table S5.** Description of OS of BMI groupings in the NIAC group (see Fig. S4B), **Table S6.** Description of OS of PLN groupings in the NIAC group (see Fig. S4B), **Table S7.** Description of CSS of PLN groupings in the NIAC group (see Fig. S4B)

**Additional file 2: Figure S1.** Propensity-score matching analysis based on follow-up duration (Box plot), (A), Distribution of different groups of patients by follow-up time before the match (B), Distribution of different groups of patients by follow-up time after 1:3 matching, **Figure S2.** Tumor staging changes in the NIAC group after matching (see Table S1), **Figure S3.** Tumor staging changes in the IAC group (see Table S2), **Figure S4.** Overall survival and cancer-specific survival from Cox proportional hazards regression analysis (see Table S3-S6), (A), Diabetes was associated with only OS (*p* = 0.004) in the IAC group. (B). BMI was only associated with OS (*p* = 0.014), and PLN was associated with both OS (p<0.001 = and CSS (*p* = 0.017) in the NIAC group, **Figure S5.** Flow diagram of the article selection process

## Data Availability

The datasets used and/or analysed during the current study available from the corresponding author on request.

## Abbreviations

ADM: adriamycin or doxorubicin
ASA: American Society of Anesthesiologists
BMI: body mass index
BUN: blood urea nitrogen
CCI: Charlson Comorbidity Index
CDDP: cisplatin
CIS: carcinoma in situ
CSS: cancer-specific survival
ECOG PS: Eastern Cooperative Oncology Group performance status
EPI: epirubicin
HCT: hematocrit
HGB: hemoglobin
IAC: intra-arterial chemotherapy
IQR: interquartile range
MMC: mitomycin C
NIAC: no-intra-arterial chemotherapy
OS: overall survival
PLN: positive lymph nodes
PLND: pelvic lymph node dissection
RC: radical
WBC: white blood cell
GC: gemcitabine + cisplatin.
ST: survival time
CSR: cumulative survival rate
