# Supplementary material for: The long-term efficacy of one-shot neoadjuvant intra-arterial chemotherapy combined with radical cystectomy versus radical cystectomy alone for bladder cancer: a propensity-score matching study
Source: BMC Urol. 2019 Nov 16;19:117. doi: 10.1186/s12894-019-0552-7 (PMC6858971; doi:10.1186/s12894-019-0552-7)
Supplement: Supplementary file 1 — Additional file 1: Table S1. Pathological staging before and after surgery in the NIAC group after matching (see Fig. S2), Table S2. Pathological staging before and after surgery in the IAC group (see Fig. S3), Table S3. Multivariable Cox proportional hazard model to estimate survival outcomes in IAC and NIAC groups, Table S4. Description of OS of diabetes groupings in the IAC group (see Fig. S4A), Table S5. Description of OS of BMI groupings in the NIAC group (see Fig. S4B), Table S6. Description of OS of PLN groupings in the NIAC group (see Fig. S4B), Table S7. Description of CSS of PLN groupings in the NIAC group (see Fig. S4B) [file 12894_2019_552_MOESM1_ESM.docx]

**Table S1.** Pathological staging before and after surgery in the NIAC group after matching (see Figure S2)

| **Pathological**  **staging** | **Clinical staging** | | | | **Total** |
| --- | --- | --- | --- | --- | --- |
|  | **T1** | **T2** | **T3** | **T4** |  |
| **T1** | 17 | 9 | 0 | 0 | 26 |
| **T2** | 0 | 21 | 4 | 0 | 25 |
| **T3** | 0 | 2 | 12 | 0 | 14 |
| **T4** | 0 | 0 | 7 | 6 | 13 |
| **Total** | 17 | 32 | 23 | 6 | 78 |

Wilcoxon signed ranks test, Z=-0.645, P=0.519

**Table S2.** Pathological staging before and after surgery in the IAC group (see Figure S3)

| Pathological  staging | Clinical staging | | | | | | Total |
| --- | --- | --- | --- | --- | --- | --- | --- |
|  | **T1** | **T2a** | **T2b** | **T3a** | **T3b** | **T4a** |  |
| T1 | 1 | 4 | 1 | 1 | 2 | 0 | 9 |
| T2a | 0 | 2 | 1 | 1 | 1 | 0 | 5 |
| T2b | 0 | 0 | 1 | 0 | 1 | 0 | 2 |
| T3a | 0 | 0 | 1 | 2 | 2 | 1 | 6 |
| T3b | 0 | 0 | 0 | 0 | 1 | 2 | 3 |
| T4a | 0 | 0 | 0 | 1 | 0 | 0 | 1 |
| Total | 1 | 6 | 4 | 5 | 7 | 3 | 26 |

Wilcoxon signed ranks test, Z=-3.157, P=0.002

**Table S3.** Multivariable Cox proportional hazards model to estimate survival outcomes in the IAC and NIAC groups

| Variables | Intra-arterial | | No intra-arterial | |
| --- | --- | --- | --- | --- |
|  | p value | RR(95%CI) | p value | RR(95%CI) |
| BMI | --- | --- | 0.015 | 0.802(0.671-0.958) |
| Diabetes | 0.029 | 14.649(1.311-163.682) | --- | --- |
| Positive lymph nodes | --- | --- | 0.000 | 7.474(2.467-22.642) |
| Smoking history | --- | --- | 0.043 | 3.388(1.040-11.038) |

**Table S4.** OS of the diabetes groupings in the IAC group (see Figure S4A)

|  | Mean ST (mo) | Medium ST (mo) | 1-year CSR (95%CI) | 5-year CSR (95%CI) | 10-year CSR (95%CI) |
| --- | --- | --- | --- | --- | --- |
| No | 116.26 | --- | 0.947(0.681-0.992) | 0.947(0.681-0.992) | 0.947(0.681-0.992) |
| Yes | 57.75 | 11.00 | 0.500(0.057-0.846) | 0.500(0.057-0.846) | --- |

ST: survival time; CSR: cumulative survival rate; OS: overall survival

**Table S5.** OS of the BMI groupings in the NIAC group (see Figure S4B)

|  | Mean ST (mo) | Medium ST (mo) | 1-year CSR (95%CI) | 5-year CSR (95%CI) | 10-year CSR (95%CI) |
| --- | --- | --- | --- | --- | --- |
| ＜25 | 101.28 | 135.00 | 0.896(0.768-0.955) | 0.761(0.608-0.861) | 0.685(0.508-0.809) |
| ≥25 | 128.55 | --- | 1.000(0.000-1.000) | 0.917(0.000-1.000) | 0.917(0.000-1.000) |

ST: survival time; CSR: cumulative survival rate; OS: overall survival; BMI: body mass index

**Table S6.** OS of the PLN groupings in the NIAC group (see Figure S4B)

|  | Mean ST (mo) | Medium ST (mo) | 1-year CSR (95%CI) | 5-year CSR (95%CI) | 10-year CSR (95%CI) |
| --- | --- | --- | --- | --- | --- |
| No | 123.05 | 135.00 | 0.967(0.873-0.992) | 0.913(0.802-0.963) | 0.879(0.740-0.946) |
| Yes | 67.43 | 49.00 | 0.833(0.000-1.000) | 0.481(0.004-0.910) | 0.361(0.007-0.809) |

ST: survival time; CSR: cumulative survival rate; CSS: cancer-specific survival; PLN: positive lymph nodes

**Table S7.** CSS of the PLN groupings in the NIAC group (see Figure S4B)

|  | Mean ST (mo) | Medium ST (mo) | 1-year CSR (95%CI) | 5-year CSR (95%CI) | 10-year CSR (95%CI) |
| --- | --- | --- | --- | --- | --- |
| No | 125.09 | 135.00 | 0.983(0.886-0.998) | 0.928(0.819-0.973) | 0.894(0.752-0.957) |
| Yes | 91.82 | --- | 0.871(0.000-1.000) | 0.586(0.000-0.967) | 0.586(0.000-0.967) |

ST: survival time; CSR: cumulative survival rate; CSS: cancer-specific survival; PLN: positive lymph nodes
