# Supplementary material for: The long-term efficacy of one-shot neoadjuvant intra-arterial chemotherapy combined with radical cystectomy versus radical cystectomy alone for bladder cancer: a propensity-score matching study
Source: BMC Urol. 2019 Nov 16;19:117. doi: 10.1186/s12894-019-0552-7 (PMC6858971; doi:10.1186/s12894-019-0552-7)

**Figure S1.** Propensity-score matching analysis based on the length of follow-up (box plot)

**
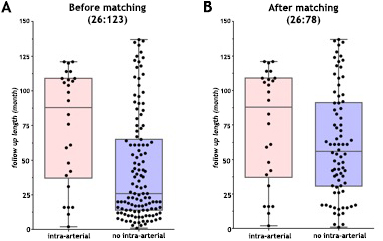
**

**Figure S2.** Changes in tumour staging in the NIAC group after matching (see Table S1).


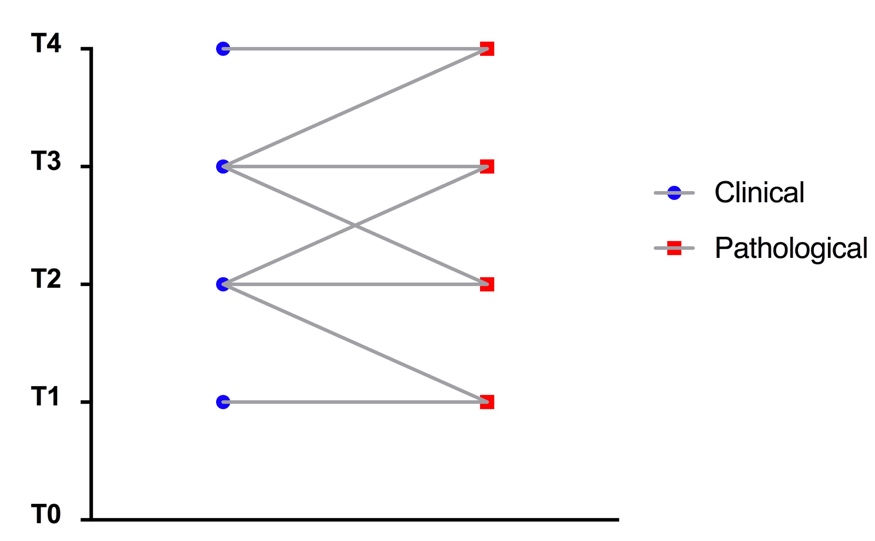


**Figure S3.** Changes in tumour staging in the IAC group (see Table S2).


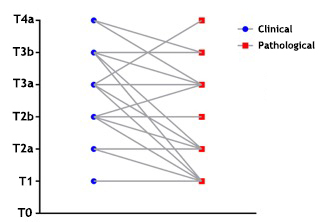


**Figure S4.** Overall survival and cancer-specific survival from Cox proportional hazards regression analysis (see Tables S4-S7).


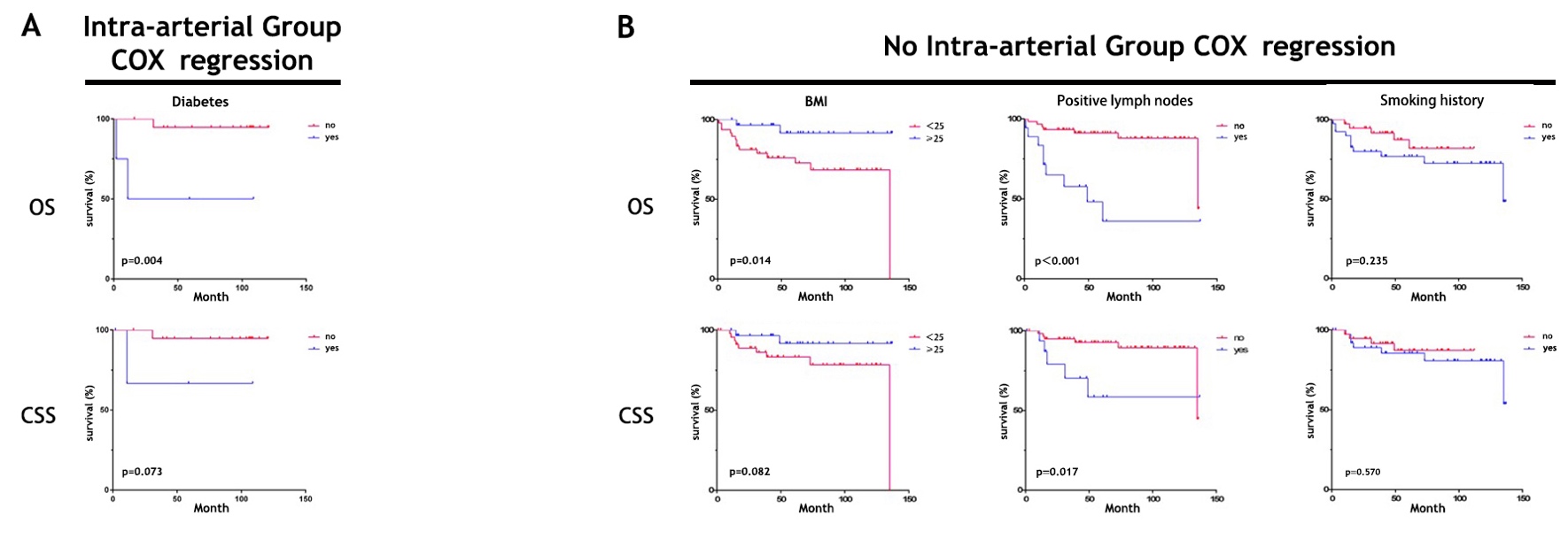


**A summary of the published neoadjuvant IAC papers, including key information on the chemotherapy regimens, is provided in Table 6.**

**Search strategy**

Search strings were used to interrogate the following databases: Medline, Embase and the Cochrane Library. Potential articles were identified using the National Library of Medicine’s Medical Subject Headings: “(((((((((((((((((((((Neoplasm, Urinary Bladder[Title/Abstract]) OR Urinary Bladder Neoplasm[Title/Abstract]) OR Neoplasms, Bladder[Title/Abstract]) OR Bladder Neoplasms[Title/Abstract]) OR Bladder Neoplasm[Title/Abstract]) OR Neoplasm, Bladder[Title/Abstract]) OR Bladder Tumors[Title/Abstract]) OR Bladder Tumor[Title/Abstract]) OR Tumor, Bladder[Title/Abstract]) OR Tumors, Bladder[Title/Abstract]) OR Urinary Bladder Cancer[Title/Abstract]) OR Cancer, Urinary Bladder[Title/Abstract]) OR Malignant Tumor of Urinary Bladder[Title/Abstract]) OR Cancer of the Bladder[Title/Abstract]) OR Bladder Cancer[Title/Abstract]) OR Bladder Cancers[Title/Abstract]) OR Cancer, Bladder[Title/Abstract]) OR Cancer of Bladder[Title/Abstract])) OR "Urinary Bladder Neoplasms"[Mesh])) AND (((((((((((((((((((((Infusion, Intra-Arterial[Title/Abstract]) OR Intra-Arterial Infusion[Title/Abstract]) OR Intra-Arterial Infusions[Title/Abstract]) OR Infusions, Intraarterial[Title/Abstract]) OR Infusion, Intraarterial[Title/Abstract]) OR Intraarterial Infusion[Title/Abstract]) OR Intraarterial Infusions[Title/Abstract]) OR Infusions, Intra Arterial[Title/Abstract]) OR Arterial Infusion, Intra[Title/Abstract]) OR Arterial Infusions, Intra[Title/Abstract]) OR Infusion, Intra Arterial[Title/Abstract]) OR Intra Arterial Infusion[Title/Abstract]) OR Intra Arterial Infusions[Title/Abstract]) OR Infusions, Regional Arterial[Title/Abstract]) OR Arterial Infusion, Regional[Title/Abstract]) OR Arterial Infusions, Regional[Title/Abstract]) OR Infusion, Regional Arterial[Title/Abstract]) OR Regional Arterial Infusion[Title/Abstract]) OR Regional Arterial Infusions[Title/Abstract])) OR "Infusions, Intra-Arterial"[Mesh])” The search was finalized on March 21, 2019.

**Selection of relevant articles**

The selection of relevant articles was conducted in two steps. As a first step, one reviewer (S.L.) excluded clearly irrelevant studies or studies not meeting the eligibility criteria based on the title and abstract. Two reviewers (S.L. and W.W.) performed the final selection based on a review of the full text of the manuscripts (Fig. S5).

**Figure S5.** Flow diagram of the article selection process


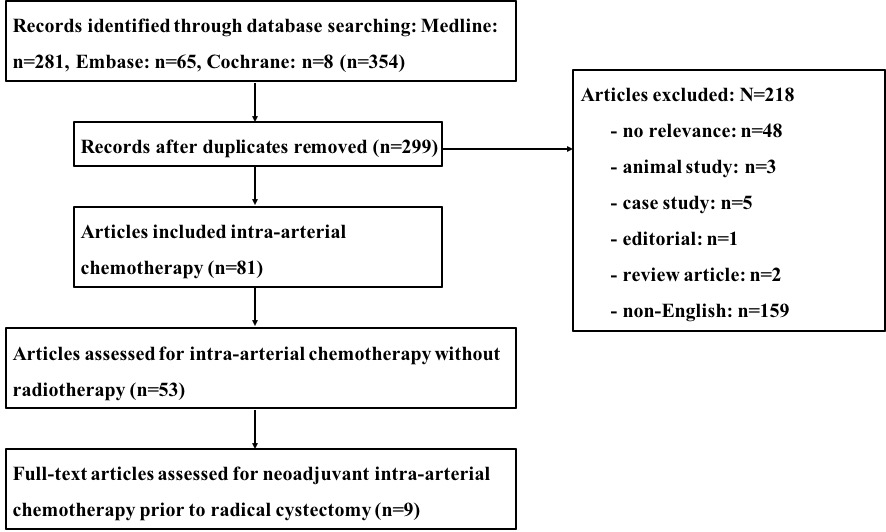

Supplement: Supplementary file 2 — Additional file 2: Figure S1. Propensity-score matching analysis based on follow-up duration (Box plot), (A), Distribution of different groups of patients by follow-up time before the match (B), Distribution of different groups of patients by follow-up time after 1:3 matching, Figure S2. Tumor staging changes in the NIAC group after matching (see Table S1), Figure S3. Tumor staging changes in the IAC group (see Table S2), Figure S4. Overall survival and cancer-specific survival from Cox proportional hazards regression analysis (see Table S3-S6), (A), Diabetes was associated with only OS (p = 0.004) in the IAC group. (B). BMI was only associated with OS (p = 0.014), and PLN was associated with both OS (p<0.001 = and CSS (p = 0.017) in the NIAC group, Figure S5. Flow diagram of the article selection process [file 12894_2019_552_MOESM2_ESM.docx]
